# Back to the future: synaesthesia could be due to associative learning

**DOI:** 10.3389/fpsyg.2014.00702

**Published:** 2014-07-07

**Authors:** Daniel Yon, Clare Press

**Affiliations:** Department of Psychological Sciences, Birkbeck, University of LondonLondon, UK

**Keywords:** synaesthesia/synesthesia, associative learning and memory, learning theory, domain specificity, experience

## Introduction

Over the last century, researchers and laypeople alike have been captivated by synaesthesia—a developmental condition present in approximately 5% of the adult population, where the presentation of an “inducer” stimulus gives rise to additional “concurrent” sensation absent from the veridical sensory world (Ward, 2013). For example, the most common variant is grapheme-color synaesthesia, where letters and digits induce concurrent sensations of color (Day, 2005).

Early theories proposed that synaesthesia resulted from associative learning between the inducer and concurrent sensory representations (Calkins, 1893; Claparede, 1903). Specifically, the inducer and concurrent stimuli have been present in a correlated, or “contingent,” fashion in the synaesthete's environment; hence presentation of the inducer alone now activates the representation of the concurrent. However, these theories have been widely rejected. A range of structural and functional mechanisms are proposed by contemporary theorists, including hyperconnectivity between brain regions (Hubbard and Ramachandran, 2005) and feedback disinhibition (Grossenbacher and Lovelace, 2001), but all are united in the assertion that “associative learning in and of itself cannot explain synaesthesia” (Marks and Odgaard, 2005).

Given recent evidence consistent with learning explanations of synaesthesia, we consider here whether the associative account may have been dismissed prematurely. We suggest that common objections to this account may be rooted in misunderstandings about what it would predict. We consider these objections in turn, highlighting how they are easily countered by contemporary learning theory. The objections are that synaesthetic experiences (1) do not reflect environmental statistics, (2) remain consistent over time, (3) cannot be trained into neurotypicals, and (4) are domain-specific. We close by describing the types of experiment that would provide the associative account of synaesthesia with the rigorous empirical assessment it deserves.

## Objections to an associative account

### Synaesthetic experience does not reflect environmental statistics

Some opponents of the associative account suggest that the seemingly bizarre correspondences synaesthetes report do not reflect the statistics of the sensory world. Deroy and Spence (2013a) make this argument when distinguishing between synaesthesia and crossmodal correspondences experienced by the typical population. They consider, for example, how the tendency of neurotypicals to match higher pitched sounds with smaller visual objects could be supported by a mechanism that tracks environmental regularities, because a note struck from a violin will be higher in pitch than one struck from a double bass, and the bark of a small dog reaches a greater frequency than that of a large one. In contrast, they propose that synaesthesia evades such an explanation because the environmental regularities it would require do not exist.

However, while the correspondences may not reflect the environmental contingencies that we all typically experience, assessing the associative account requires knowledge of the sensory environment of a particular individual across their developmental history. In fact, in contrast with the view of Deroy and Spence (2013a), Witthoft and Winawer (2013) recently reported that the particular mappings between colors and letters of 11 synaesthetes may be explained with reference to toys with which they interacted as children—such as colored magnets depicting alphanumeric characters. Such studies join an existing body of work suggesting that synaesthetic mappings may in fact reflect past (or present) environmental contingencies (e.g., Simner et al., 2005; Witthoft and Winawer, 2006; although see Rich et al., 2005).

Therefore, synaesthetic experience may reflect the statistics of the sensory environment in which synaesthetes developed even if it does not correspond to statistical relationships in the current environment of the majority.

### Consistency of synaesthetic experience

It has also been argued that if synaesthetic experience arises due to environmental correlations between inducer and concurrent stimuli, then exposure to the typical environment—when inducer-concurrent relationships are no longer present—should give rise to “reversal learning” of the association (Gray et al., 2006): If associations are learned through experience, they should also be *unlearned* through experience. Therefore, it is proposed that the high consistency of synaesthetic experience is evidence against the associative account (although see Simner et al., 2009; Simner and Bain, 2013; Meier et al., 2014).

This objection displays some misunderstanding of the associative literature, which does in fact predict that synaesthetic experience should exhibit high consistency. To understand this prediction, we must consider how reversal learning is thought to operate. True unlearning, or “extinction” of associations, operates over vast timescales if it occurs at all (Baeyens et al., 1995). “Unlearning” more typically involves the establishment of new, alternative associations. As with the formation of first-learned associations, such second-learned associations will only be formed if there is a systematic, i.e., contingent, new relationship in the environment (Dickinson and Charnock, 1985). Furthermore, while first-learned associations formed with novel stimuli generalize well to other contexts, subsequently acquired associations, which give rise to ambiguity, demonstrate contextual specificity (Bouton, 1993, 1994; Nelson, 2002).

Consider a grapheme-color synaesthete who associates the letter “A” with the color red. The formation of a new competing association would only occur if there is a systematic pairing between “A” and another stimulus. If this does occur, for example, “A” is contingently paired with the color black, the synaesthete will still maintain the first-learned “A”-red association alongside context-dependent “A”-black associations. Thus, testing in contexts alternative to that in which the “A”-black association was learned (e.g., the dark computer cubicles at universities where many synaesthetes are assessed) will still highlight only context-independent A-red associations.

Therefore, an associative account in fact predicts that first-learned associations mediating synaesthetic experience should influence perception with a high degree of consistency, although consistency will be determined by the timecourse of relevant learning in combination with the similarity of training and test contexts for second-learned associations.

### Synaesthesia cannot be “trained into” neurotypicals

Others propose that if synaesthesia is generated by learned associations, training in neurotypicals should generate similar experiences. The associative account is often rejected on the basis that some studies fail to find a change in the neurotypical participant's phenomenology after such training (see Deroy and Spence, 2013b; Rothen and Meier, 2014 for reviews).

Notably training studies *have* been successful in inducing behavioral changes that are akin to those seen in synaesthesia. For example, previous researchers have reported the “synaesthetic-Stroop effect,” where synaesthetes are slower to respond to graphemes (Dixon et al., 2000; Nikolic et al., 2007) or tones (Ward et al., 2006) when they are presented with colors that are incongruent with the concurrent they typically elicit. Exposing neurotypical subjects to arbitrary pairings in the laboratory generates similar interference effects post-training (e.g., Howells, 1944; Rothen et al., 2011; Colizoli et al., 2012; Kusnir and Thut, 2012).

Even if the phenomenology of synaesthetes differs from that of a neurotypical after training, it does not follow that distinct mechanisms mediate the effects. Phenomenological sensation that a concurrent stimulus is present is likely to be determined by the strength of activation of the perceptual representation, which is likely to differ in the two scenarios. Namely, the amount of experience presented in a typical training study is dwarfed by that of a child in their developmental environment (Cohen Kadosh et al., 2005), and second-learned associations—which likely mediate neurotypical training effects in adults—often require more exposure before acquisition (Myers et al., 2000). Lesser opportunity to learn would result in a weaker association (Hull, 1943). Therefore, any differences in phenomenology may be due to imbalanced strength of “inducer”-“concurrent” associations, resulting in lesser activation of “concurrent” representations in neurotypical training studies (note that similar explanations address the differences in longevity of behavioral changes; Deroy and Spence, 2013b).

In summary, to test whether experiences akin to those of a synaesthete can be trained into a neurotypical, future studies should examine how the induction of stronger associations modulates effects (Rothen et al., 2011).

### Domain-specificity

The associative account proposes that the learning mechanisms generating synaesthetic experience operate across domains. The final common objection suggests that this account “fails… because it cannot address… why only specific classes of stimuli are able to induce synaesthesia” (p. 4, Ramachandran and Hubbard, 2001). For example, many synaesthetes report cultural artifacts as inducers—like letters, musical notes and words—with fewer reporting correspondences between, say, touch and taste. One might argue that associative explanations fail to account for why some variants of synaesthesia are so common, and others so rare (Marks and Odgaard, 2005).

When considering this objection it is first important to note that while certain types of synaesthesia are more common, it is likely that individual synaesthetes will present with multiple types (Ramachandran and Hubbard, 2001). Second, while the learning mechanisms generating the condition may be domain-general, it does not follow that effects should be present across all domains. For example, to learn about stimuli they must be processed, therefore environmental stimuli to which children are encouraged to attend for lengthy periods—such as letters when learning to read—would be recipients of a learning advantage (Mackintosh, 1975). Furthermore, while associative theories analyze general processes of excitation and inhibition between cognitive representations, they do not deny the existence of certain anatomical constraints on generality; such that some associations may be easier to form if representations are located in neighboring cortical regions (Bargary and Mitchell, 2008). However, acknowledging such constraints does not detract from the domain-generality of the mechanism proposed; it is domain-general learning mechanisms that are atypical, not the anatomical constraints on generality (note that under this account any genetic contributions would act solely upon the operation of the learning mechanisms).

Therefore, a domain-general mechanism can generate effects that do not operate across all domains.

## Testing the associative account

Given that objections to the associative account have been arguably premature, we propose that it should now receive rigorous empirical assessment.

To date, learning accounts have been assessed by either considering whether contingencies existed in the developmental environment that could have generated inducer-concurrent associations or conducting training studies in neurotypicals. These studies provide an excellent starting point for testing the plausibility of the associative account, and recent rigorous examinations provide evidence consistent with learned origins of the associations (Witthoft and Winawer, 2013; Rothen and Meier, 2014). However, there are two problems with using these strategies in isolation. First, the former rests heavily on self-report. It is likely that many of the contingencies of interest would have been present during the period of childhood amnesia (Bauer and Larkina, 2013), and even if they were present later in life it is often difficult to assess these claims systematically; for example, whether contingencies were strong enough to produce learning, and conversely whether absence of evidence is really evidence of absence. Second, neither approach can identify the precise mechanism by which synaesthetic experience emerges—namely what is special about synaesthetes, and why, given the same environmental input, typical individuals do not develop such unusual experiences.

We suggest that the time has come to investigate systematically whether and how learning mechanisms differ in synaesthetes. Some studies already raise the possibility that grapheme-color synaesthetes possess enhanced visual learning abilities (Rothen et al., 2012, 2013; Pritchard et al., 2013; Ward et al., 2013; although see Gross et al., 2011). However, while advantages may be due to intrinsic differences in associative mechanisms, it is equally plausible that the experience of having synaesthesia causes these individuals to direct more attention to the modality of their inducer/concurrent (see Mackintosh, 1975). More targeted tests looking specifically at associative processes in synaesthetes (e.g., the magnitude of blocking, contingency, context, and latent inhibition effects, Dickinson, 1981) relative to neurotypicals, across a range of domains, and without competition from the synaesthetic domain (domains will need to be selected carefully to avoid confounding the causes and effects of synaesthesia), are thus needed to investigate the associative account. For example, the atypical correspondences seen in synaesthesia could be accounted for if this group are “fast learners”—requiring fewer pairings or weaker contingencies to form associations between stimulus features.

## Conclusion

The theory that synaesthesia emerges through associative learning may have been dismissed prematurely. Objections to this account frequently miss the subtleties of contemporary associative theory. Therefore, we propose that it is time to re-consider this account as a viable explanation of synaesthesia and give it the rigorous empirical assessment that it deserves.

### Conflict of interest statement

The authors declare that the research was conducted in the absence of any commercial or financial relationships that could be construed as a potential conflict of interest.
